# Exploding Totem in Art and Biology

**DOI:** 10.3201/eid1301.000000

**Published:** 2007-01

**Authors:** Polyxeni Potter

**Affiliations:** * Centers for Disease Control and Prevention, Atlanta, Georgia, USA

**Figure:**
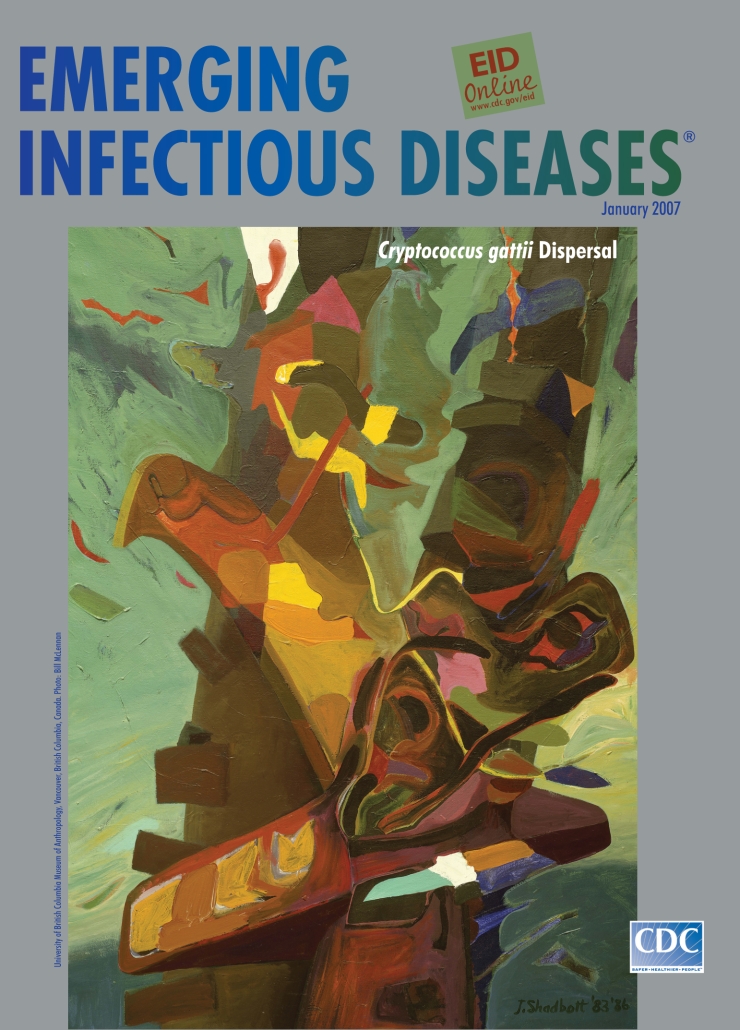
**Jack Shadbolt (1909−1998). Toward Totem (1983−1986).** Acrylic paint on canvas (177.7 cm × 127.4 cm). University of British Columbia Museum of Anthropology, Vancouver, British Columbia, Canada. Photo: Bill McLennan

“What could be better than dying in the place you spent your life working…?” Doris, Jack Shadbolt’s wife, mused, as she and friends moved his bed into the studio during the final days of his life. “When I can’t paint, I don’t want to live,” he had said (*1*). Around the bed was Blue Breaking, a triptych he had painted, filled with shadowy forms, “like butterflies’ footprints.” A few days later, Shadbolt died of heart failure in Burnaby, British Columbia, where he had lived for more than 50 years.

Born in Shoeburyness, England, Shadbolt grew up in Victoria, Canada, to become an iconic figure, not only as artist but also as author and teacher. He began painting early in life, copying scenes from calendars with his father, a craftsman and amateur painter. He studied at Victoria College and Normal School, but his career took off when he started sketching the Victoria area with his friend Max Maynard, who introduced him to Emily Carr^1^ (*2*). “To come under the immediate spell of a famous artist one admires tremendously and, at the same time, encounters personally in one’s own local community,” he wrote, “is the most compelling influence for an artist (*3*).”

Shadbolt traveled widely. He studied in London and Paris (1937−1938) and at the Art Students’ League in New York City (1948). He became interested in the work of Pablo Picasso and Joan Miró and is linked to these and other modern masters. During World War II, at work with the Canadian Army War Artists in London, he sketched war scenes, barbed wire fences, watchtowers, and the bleak life of prisoners. This brush with darkness affected his individual brand of modernism, “…when the bomb blows the building apart it abstracts it, the pieces fall back together again and you get a memory image of what was there but vastly altered and psychologically made infinitely more intense than the original thing” (*2*). Yet, throughout his long career, his main inspiration was the regional West Coast culture and indigenous arts, “The nearest symbolic mythology at hand” (*3*).

“The shapes of knowledge are always ineluctably local,” wrote anthropologist Clifford Geertz (*4*). The artist draws strength from the community. The more intensely William Faulkner focused on his unpronounceable county in Mississippi, the more intelligible he became to readers in all corners of the world (*5*). Shadbolt knew that his authenticity was linked to his region.

Originated in his initial contact with Emily Carr, his complex relationship with Coastal Indian art was described in a journal entry, “The Indian mode of expressing things from the inside out, out of deep interior identification with the spirit of the image portrayed, gave me my inventive impetus as well as helping me with my personal mode of abstraction” (*6*).

Along with a New York group that included Barnett Newman, Mark Rothko, Adolph Gottlieb, and Jackson Pollock, Shadbolt saw tribal art as “timeless and instinctive, on the level of spontaneous animal activity, self-contained, unreflective, private, without dates and signatures, without origins or consequences except in the emotions” (*7*). This group, the primitivists of the 1940s, promoted a genre that was not, as it appeared, about tribal art but about what Shadbolt called “the act of art” (*8*).

“Creating is not an exercise in aesthetics but an act of sensory discovery. Having made a right move, one parlays it to the next move, and the next, and the next, until the (visual) reality grows into an unexpected thing under one’s hands” (*3*). Shadbolt explains the process, “I have very little of the mystic in me, but if there is any it is in my rapture when, in the work process, that state is attained where everything seems a part of everything, each form existing for itself but not being itself until it also answers every other form in a perfect coordination of reciprocal intent….The ‘whole is greater than the sum of the parts’ is what art is all about” (*3*).

Caught in a blur of vibrant color, Toward Totem, on this month’s cover, seems a mirage of its original intent. In the forefront, jumbled parts—a raven’s beak, a glaring eye—jut off a degenerating totem pole. Superimposed on a solid diagonal on the left, the explosive image lights up a new pole, defined by the glare on surrounding flat surfaces. The abstraction of an old artifact, Shadbolt’s fantastic modern equivalent sprouting in the background seems unconstrained by tradition, animal, clan, or taboo, even as its emotional charge betrays the artist’s affection for the original.

In traditional terms, “totem,” most often an animal, or class of animals, has a special relationship with the clan. Common ancestor, guardian spirit, and helper of the clan, totem is dangerous to outsiders but recognizes and spares its own, thus earning emblematic status as family crest, symbol, or historical record. Abstracting the essence but not the naturalistic image of Northwest Coast totem, Shadbolt created a modern form, which unbeknown to him, may be closer to biological reality than the original.

Microorganisms are the microbial equivalent of totem. Though originating with and in general supportive of the clan, they do not linger to protect their own. Under evolutionary, ecologic, and societal pressures, they disperse. *Cryptococcus gattii* is a case in point. Infections away from Vancouver Island (initial focus), in other parts of the Pacific Northwest, indicate that away from the clan, the organisms have been spreading to humans and animals through common agricultural practices (*9*).
